# Generation of iVero.219-mcRTA: a doxycycline-inducible high-titer KSHV producer cell line with multicopy *orf50* integration

**DOI:** 10.3389/fcimb.2026.1760149

**Published:** 2026-04-13

**Authors:** Li Wang, Xueli Wang, Qiyan Chen, Junli Wu, Yanli Xu, Jinzhong Wang, Ying Wang

**Affiliations:** 1 TEDA Institute of Biological Sciences and Biotechnology, Nankai University, Tianjin, China; 2 Key Laboratory of Molecular Microbiology and Technology, Ministry of Education, Tianjin, China; 3 Tianjin Key Laboratory of Microbial Functional Genomics, Tianjin, China

**Keywords:** doxycycline-inducible, iVero.219-mcRTA, KSHV, multicopy integration, RTA

## Abstract

Kaposi’s sarcoma-associated herpesvirus (KSHV) is an oncogenic virus whose reactivation from latency to lytic replication is orchestrated by the replication and transcription activator (RTA) encoded by *orf50*. Doxycycline (DOX)-inducible RTA expression is commonly used in engineered cellular models to trigger this reactivation and produce infectious virus. However, this approach often requires co-treatment with histone deacetylase inhibitors (HDACi), such as sodium butyrate (SB), whose pleiotropic epigenetic effects can obscure experimental interpretation. To address this limitation, we generated the iVero.219-mcRTA polyclonal cell line by sequential lentiviral transduction using vectors with distinct antibiotic selection markers, achieving stable multicopy integration of the inducible RTA cassette, with total copy number reaching approximately 150 per cell as determined by droplet digital PCR. Upon DOX induction alone, 51.6% of cells expressed RFP, indicating efficient reactivation. qPCR quantification of viral DNA in the supernatant showed (5.7 ± 0.1) × 10^4^ genome copies/mL, and infection assays in 293T cells yielded an infectious titer of (2.4 ± 0.1) × 10^4^ IU/mL based on Poisson distribution analysis of serial dilutions. Supplementation with SB enhanced these values to (1.5 ± 0.1) × 10^5^ genome copies/mL and (7.5 ± 0.2) × 10^4^ IU/mL, respectively. Notably, the supernatant induced with DOX alone, once concentrated, achieved an infectious titer of (2.1 ± 0.1) × 10^5^ IU/mL, without the need for HDACi. Collectively, the iVero.219-mcRTA system enables robust, high-titer KSHV production using DOX alone, providing a simplified and HDACi-independent tool that will facilitate future KSHV research.

## 1 Introduction

Kaposi’s sarcoma-associated herpesvirus (KSHV), also known as human herpesvirus 8, is an oncogenic double-stranded DNA virus of the Gammaherpesvirinae subfamily (Chang et al., 1994). It is the etiological agent of Kaposi sarcoma, primary effusion lymphoma, and multicentric Castleman disease, and is particularly prevalent among AIDS patients (Cesarman et al., 1995; Soulier et al., 1995; Dupin et al., 1999; Ganem, 2010). Like other herpesviruses, KSHV exhibits a biphasic life cycle consisting of latent and lytic stages. Latency allows lifelong persistence of the viral episomal genome without particle production, whereas the lytic cycle drives active replication, virion release, and disease progression (Broussard and Damania, 2020; Losay and Damania, 2025).

The transition from latency to lytic replication, known as reactivation, is a pivotal event in KSHV pathogenesis and a major target for therapeutic intervention (Verma and Robertson, 2003; Purushothaman et al., 2015; Broussard and Damania, 2020; Sandhu and Damania, 2022). This process is orchestrated primarily by the replication and transcription activator (RTA), an immediate-early protein encoded by the viral *orf50* gene. As the master lytic switch protein, RTA is both necessary and sufficient to initiate the full lytic program through directly transactivating a series of downstream viral promoters (Lukac et al., 1999; Pavlova et al., 2003; Xu et al., 2005). Upon reaching a critical threshold, RTA competitively binds to RBP-Jκ, a host transcriptional repressor and the major binding partner of latency-associated nuclear antigen during latency, thereby relieving latent transcriptional repression and promoting chromatin remodeling. This enables RTA to bind specific response elements within the KSHV genome and activate early lytic genes (Guito and Lukac, 2015). The ensuing transcriptional cascade triggers viral DNA replication, culminating in the assembly and release of infectious progeny virions (Sun et al., 1999).

Several cell models have been developed to study KSHV reactivation and infectious virion production. Among them, primary effusion lymphoma (PEL)-derived B cell lines can be reactivated using pleiotropic chemical inducers. However, their high rate of spontaneous reactivation generates substantial background lytic activity and thereby obscures stimulus-specific signals (Renne et al., 1996; Sun et al., 1999; Kati et al., 2013; Dollery et al., 2014). In contrast, most KSHV-infected adherent cell lines, such as iSLK.219 and iTIME.219, utilize tetracycline- or doxycycline (DOX)-inducible systems to achieve stringent control over lytic reactivation, exhibiting minimal background leakage in the absence of induction. Because lytic induction depends on random genomic integration of a single-copy *orf50* transgene, RTA expression is often insufficient to overcome host epigenetic barriers (Myoung and Ganem, 2011; Dollery et al., 2021), resulting in low yields of infectious progeny and requiring labor-intensive monoclonal screening.

The Vero.219 cell line represents a classic and widely adopted model system, generated by infecting Vero cells with the recombinant rKSHV.219 virus harboring an RFP/EGFP dual fluorescent reporter system (Vieira and O'Hearn, 2004). Although puromycin selection in this system aids in maintaining viral genome stability, its reliance on exogenous RTA expression and high-dose chemical inducers for reactivation introduces considerable practical constraints. Transient expression of exogenous RTA typically yields low viral titers and considerable batch-to-batch variability (Bechtel et al., 2003; DeCotiis et al., 2017). Chemical inducers, such as the broad-spectrum protein kinase C activator 12-O-tetradecanoylphorbol-13-acetate (TPA) and histone deacetylase inhibitors (HDACi) including sodium butyrate (SB) and valproic acid (VPA), can only partially activate latent virus (Tovey et al., 1978; Renne et al., 1996; Shaw et al., 2000; McAllister et al., 2005). Even combined treatment with TPA and SB generally induces viral titers consistently below 1 × 10^4^ IU/mL (Vieira and O'Hearn, 2004). Additionally, these compounds frequently cause substantial cytotoxicity and profoundly disturb host cell chromatin architecture as well as signaling pathways, necessitating additional purification steps to eliminate these effects and obtain viral supernatants free of confounding factors (Gao et al., 2003).

To overcome these limitations, we generated the iVero.219-mcRTA polyclonal cell line by sequential lentiviral transduction of Vero.219 with Lenti-X Tet-One vectors carrying DOX-inducible RTA and distinct antibiotic markers. These iVero.219-mcRTA cells (mc, multicopy) stably integrate multiple copies of the *orf50* transgene and support potent yet tightly controlled RTA induction with DOX alone, thereby reactivating KSHV and yielding infectious virions without HDACi. Although SB co-treatment can further enhance output, concentrated supernatants from DOX-only cultures already provide high-titer virus stocks. Owing to its simple induction, high viral yields, and ease of handling, the iVero.219-mcRTA cell line represents a robust and versatile tool for high-titer rKSHV.219 production and for mechanistic studies of KSHV reactivation and pathogenesis.

## 2 Materials and methods

### 2.1 Plasmids

The lentiviral vectors pLVX-TetOne-HygR and pLVX-TetOne-NeoR, the packaging plasmid psPAX2, and the envelope plasmid pMD2.G were obtained from Miaoling Plasmid (Wuhan, China). To generate pLVX-TetOne-BleoR, the *Sh ble* gene, which confers resistance to zeocin, was amplified by PCR from pcDNA3.1-zeo using primers BleoR-F and BleoR-R. The amplified fragment was cloned into the *Mlu* I and *Hpa* I sites of pLVX-TetOne-HygR, replacing the hygromycin-resistance (HygR) gene. To enable directional cloning of the KSHV *orf50* gene, which encodes RTA, a synthetic multiple cloning site (MCS) was generated by annealing complementary oligonucleotides MCS-F and MCS-R and subsequently introduced into the existing *Bam* HI and *Eco* RI sites of each vector. The KSHV *orf50* gene was then amplified from Vero.219 total DNA with primers ORF50-F and ORF50-R and directionally inserted into each vector using the *Bam* HI site and the *Not* I site within the engineered MCS, yielding the final constructs pLVX-TetOne-HygR-RTA, pLVX-TetOne-NeoR-RTA, and pLVX-TetOne-BleoR-RTA. All oligonucleotides used in this study were purchased from Azenta Life Sciences; their sequences are provided in **Supplementary Table 1**. Final constructs were verified by DNA sequencing.

### 2.2 Cell culture

HEK 293T, Vero.219, and iVero.219-mcRTA cells were maintained in Dulbecco’s modified Eagle medium (DMEM) supplemented with 10% fetal bovine serum (Sangon Biotech) and 1% penicillin-streptomycin at 37 °C with 5% CO_2_. To retain the recombinant rKSHV.219 genome, Vero.219 and iVero.219-mcRTA cells were cultured under continuous selection with 5.0 µg/mL puromycin (Vieira and O'Hearn, 2004).

### 2.3 Preparation of recombinant lentiviruses

Recombinant lentiviruses were produced by co-transfecting 293T cells with the transfer plasmids pLVX-TetOne-HygR-RTA, pLVX-TetOne-NeoR-RTA, or pLVX-TetOne-BleoR-RTA, along with the packaging plasmid psPAX2 and the envelope plasmid pMD2.G, using polyethylenimine (MW 40000, YEASEN) as the transfection reagent. Viral supernatants were collected at 48 h and 72 h post-transfection, followed by centrifugation at 1500 × *g* for 10 min at 4 °C to remove cellular debris. The clarified supernatants were then filtered through 0.45 μm PVDF membranes. Lentiviral particles were concentrated by centrifugation at 15000 × *g* for 4 h at 4 °C, and the resultant pellet was resuspended in ice-cold DMEM at 1/100 volume. The concentrated viruses were then either used immediately or aliquoted and stored at −80 °C for future use.

### 2.4 Generation of iVero.219-mcRTA cell line

Vero.219 cells were engineered to carry multiple stably integrated *orf50* copies for sustained RTA overexpression *via* sequential lentiviral transduction. Three RTA-encoding lentiviruses harboring neomycin, hygromycin, or zeocin selectable markers were delivered in successive rounds. Each virus was used for two rounds of transduction with a 48 h interval; each round included spinoculation at 800 × *g* for 1 h at room temperature in the presence of 5 μg/mL polybrene (Beyotime). Following the second transduction with the neomycin-resistant virus, cells were selected with 400 µg/mL G418 for 7 days. The same two-round protocol was repeated with the hygromycin-resistant virus, and cells were then co-selected for 7 days with 400 µg/mL G418 and 300 µg/mL hygromycin. Finally, two rounds of transduction were performed with the zeocin-resistant virus, after which antibiotic concentrations were raised to 600 µg/mL G418, 500 µg/mL hygromycin, and 300 µg/mL zeocin, and cells were maintained under continuous co-selection conditions for 14 days to ensure stable genomic integration of multiple *orf50* copies, yielding the polyclonal iVero.219-mcRTA line. The established iVero.219-mcRTA cells were maintained under continuous antibiotic selection (puromycin, G418, hygromycin, and zeocin) to ensure stable retention of multicopy *orf50* integration.

### 2.5 Detection of *orf50* gene integration

Genomic DNA was extracted from lentiviral-transduced Vero.219 cells using the Ezup Column Animal Genomic DNA Purification Kit (Sangon Biotech) according to the manufacturer’s instructions. PCR amplification of the lentiviral integration marker WPRE, the *orf50* gene, and the internal control *gapdh* gene was performed with the primers listed in **Supplementary Table 1**. To quantify the integrated and total copy numbers of the *orf50* gene per cell, quantitative PCR (qPCR) was conducted using the UltraSYBR Mixture (CoWin Biosciences) on an Applied Biosystems QuantStudio 5 system. All primer pairs showed 90-110% efficiency with R²> 0.99. Absolute quantification was performed using standard curves generated from calibration standards of known concentrations. Normalization of the *orf50* and WPRE copy numbers to the housekeeping gene *gapdh* enabled the determination of copy number per cell.

Genomic DNA samples were also submitted to Sangon Biotech for droplet digital PCR (ddPCR) analyses using the AD16 automated ddPCR system (Pilot Gene Technology). Absolute concentrations of WPRE and *orf50* were calculated using the Poisson distribution algorithm with the Pilot Gene analysis software, and the copy number of each target per cell was normalized to the monkey reference gene *rpp30* (2 copies per Vero cell; Zhang et al., 2025). Primers and probes for WPRE, *orf50* (Sharma-Walia et al., 2014), and *rpp30* are listed in **Supplementary Table 1**.

### 2.6 SDS-PAGE and Western blot

Vero.219 or iVero.219-mcRTA cells were seeded in 12-well plates at a density of 1 × 10^5^ cells per well. After reaching 80% confluency the following day, cells were treated with DOX and/or SB for 72 h to induce the expression of lytic genes. Cells were then washed twice with phosphate-buffered saline and lysed in 1× SDS sample buffer. Lysates were sonicated on ice and boiled at 100 °C for 5 min. Protein separation and immunoblotting were performed as previously described (Qin et al., 2010). Briefly, proteins were resolved by SDS-PAGE using a 10% separating gel and a 4% stacking gel, and subsequently transferred to a PVDF membrane (GE Healthcare). After blocking with 5% non-fat milk (Sangon Biotech), the membrane was incubated with primary antibodies against RTA (Qin et al., 2010), ORF45, ORF57 and β-Tubulin (Sigma-Aldrich), followed by incubation with HRP-conjugated secondary antibody (Santa Cruz Biotechnology). Protein bands were visualized using SuperSignal West Pico Chemiluminescent Substrate (Thermo Fisher Scientific) and Amersham Imager 600.

### 2.7 Production and titration of KSHV progeny virus stocks

To induce lytic replication for the production of rKSHV.219, Vero.219 or iVero.219-mcRTA cells were treated with DOX, SB, or their combination at various concentrations. Supernatants were collected 72 h post-induction, centrifuged at 1500 × *g* for 10 min at 4 °C, and filtered through 0.45 μm PVDF membranes. To further increase the viral titers, the supernatants were centrifuged at 15000 × *g* for 4 h at 4 °C, and the resultant pellet was resuspended in ice-cold DMEM at 1/100 volume.

Viral supernatants were pre-treated with DNase I (Thermo) to remove free DNA contaminants. Following heat inactivation of the enzyme, viral genomic DNA was extracted using the Blood Genomic DNA Extraction Kit (TIANGEN) according to the manufacturer’s protocol. The KSHV genomic copy number was determined by qPCR using primers (**Supplementary Table 1**) targeting the *k9* gene (Dong et al., 2021). Absolute viral copy numbers were determined based on a standard curve generated from serially diluted *k9*-containing calibration standards of known concentrations.

For KSHV infection assays, 293T cells were seeded in 24-well plates at 1 × 10^5^ cells/well. The following day, 1 mL of filtered virus-containing supernatant or 200 μL 100× concentrated supernatant was added to each well. Plates were centrifuged at 1500 × g for 1 h at 25 °C and then incubated at 37 °C for 2 h (Yoo et al., 2008). The inoculum was subsequently replaced with complete medium, and cells were incubated for 24 h. For titration of infectious virions, 293T cells were seeded in 96-well plates at 1 ×10^4^ cells/well 24 h prior to infection. Serial dilutions of virus in serum-free DMEM were added to cells, followed by spinoculation as described above. Infectious titers were calculated using Poisson distribution statistics within the linear range of infection (10-20% GFP-positive cells). Fluorescence images were acquired using a ZEISS Axio Observer 7 microscope with a 20× objective and processed with ZEN software. The percentage of EGFP-positive cells was quantified by flow cytometry (Kati et al., 2015).

### 2.8 Flow cytometry

RFP and/or EGFP expression was analyzed by flow cytometry in Vero.219, iVero.219-mcRTA, or rKSHV.219-infected 293T cells. DOX and/or SB induced cells and uninduced control cells were harvested and centrifuged at 300 × g for 3 min. The pellet was resuspended in DMEM for immediate analysis. Fluorescence signals were acquired on a BD FACSymphony™ A5 Flow Cytometer. Data were processed with FlowJo software, with FACS gating set so that <0.1% of uninduced control cells fell within the positive region.

### 2.9 Statistical analysis

Statistical analyses were performed using GraphPad Prism. Data are presented as mean ± standard deviation (SD) from at least three independent repeat experiments. One-way ANOVA followed by Dunnett’s multiple comparisons test was used for comparisons against a single control group, and Tukey’s multiple comparisons test was used for all pairwise comparisons. A *P*-value of less than 0.05 was considered statistically significant.

## 3 Results

### 3.1 Production of lentiviral particles with distinct antibiotic resistance for sequential *orf50* transduction

**Figure 1:**
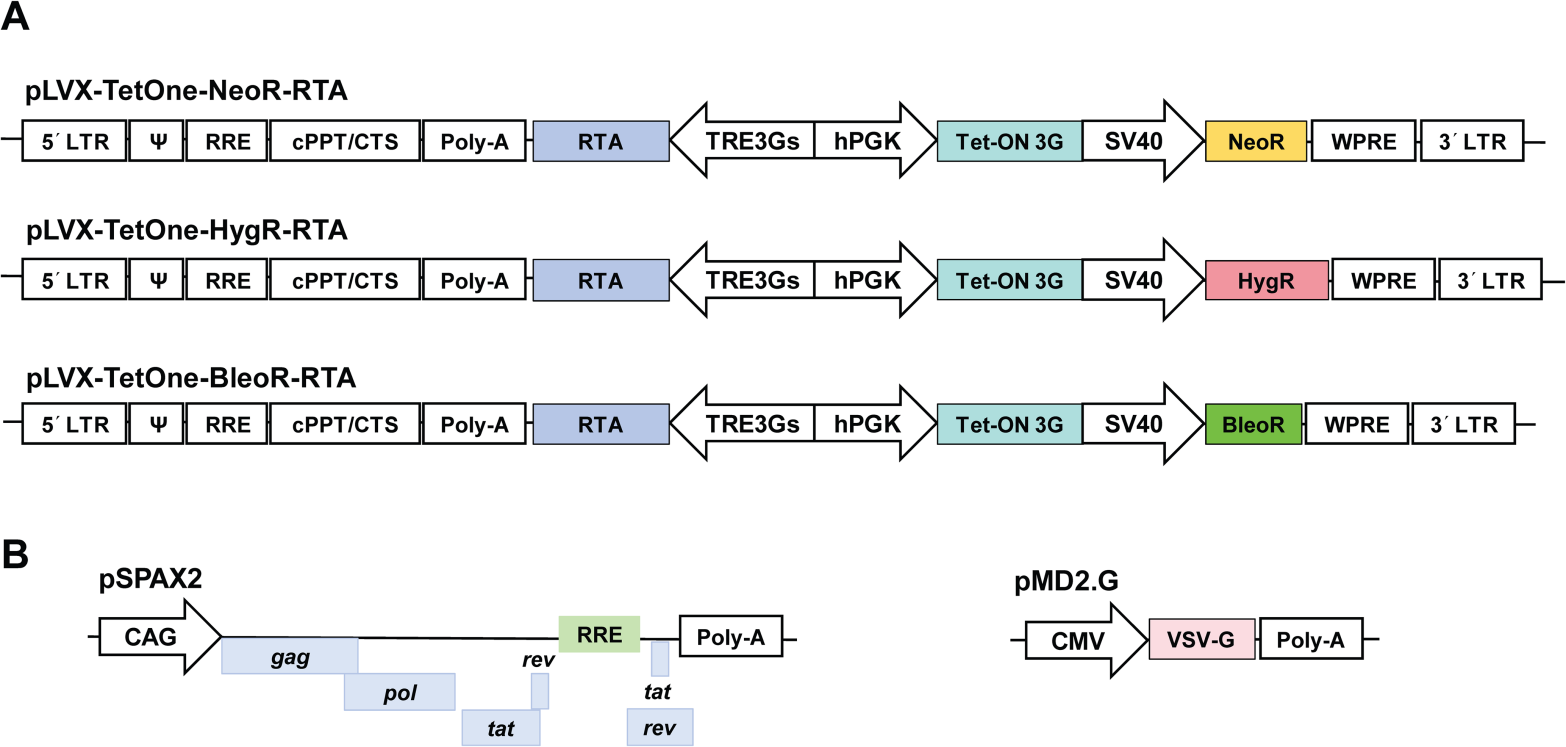
Schematics of Tet-On inducible lentiviral vectors. **(A)** Three pLVX-TetOne transfer vectors, each carrying full-length *orf50* and a neomycin, hygromycin, or zeocin resistance cassette. DOX binding to the Tet-On 3G transactivator induces its recruitment to the *P*_TRE3G_ promoter, driving RTA expression. **(B)** psPAX2 and pMD2.G components of the three-plasmid lentiviral packaging system.

Lentiviral vectors were constructed using the all-in-one pLVX-TetOne system to enable DOX-inducible expression of the KSHV *orf50* gene, which encodes RTA. The *orf50* expression is under the control of the *P*_TRE3G_ promoter, which is activated by the constitutively expressed Tet-On 3G transactivator upon DOX binding (Loew et al., 2010). To facilitate the selection of cells containing multicopy integrations of the *orf50* gene, three vectors were generated, each carrying a distinct antibiotic resistance gene for selection under G418 (neomycin), hygromycin, or zeocin. The resulting constructs were designated pLVX-TetOne-NeoR-RTA, pLVX-TetOne-HygR-RTA, and pLVX-TetOne-BleoR-RTA (**Figure 1A**).

Recombinant lentiviral particles were produced in 293T cells using the classic three-plasmid system, which comprised one of these three constructs as the transfer vector, the packaging plasmid psPAX2, and the envelope plasmid pMD2.G to supply the vesicular stomatitis virus G glycoprotein (**Figure 1B**).

### 3.2 Generation of iVero.219-mcRTA cell line harboring multicopy DOX-inducible *orf50*

**Figure 2:**
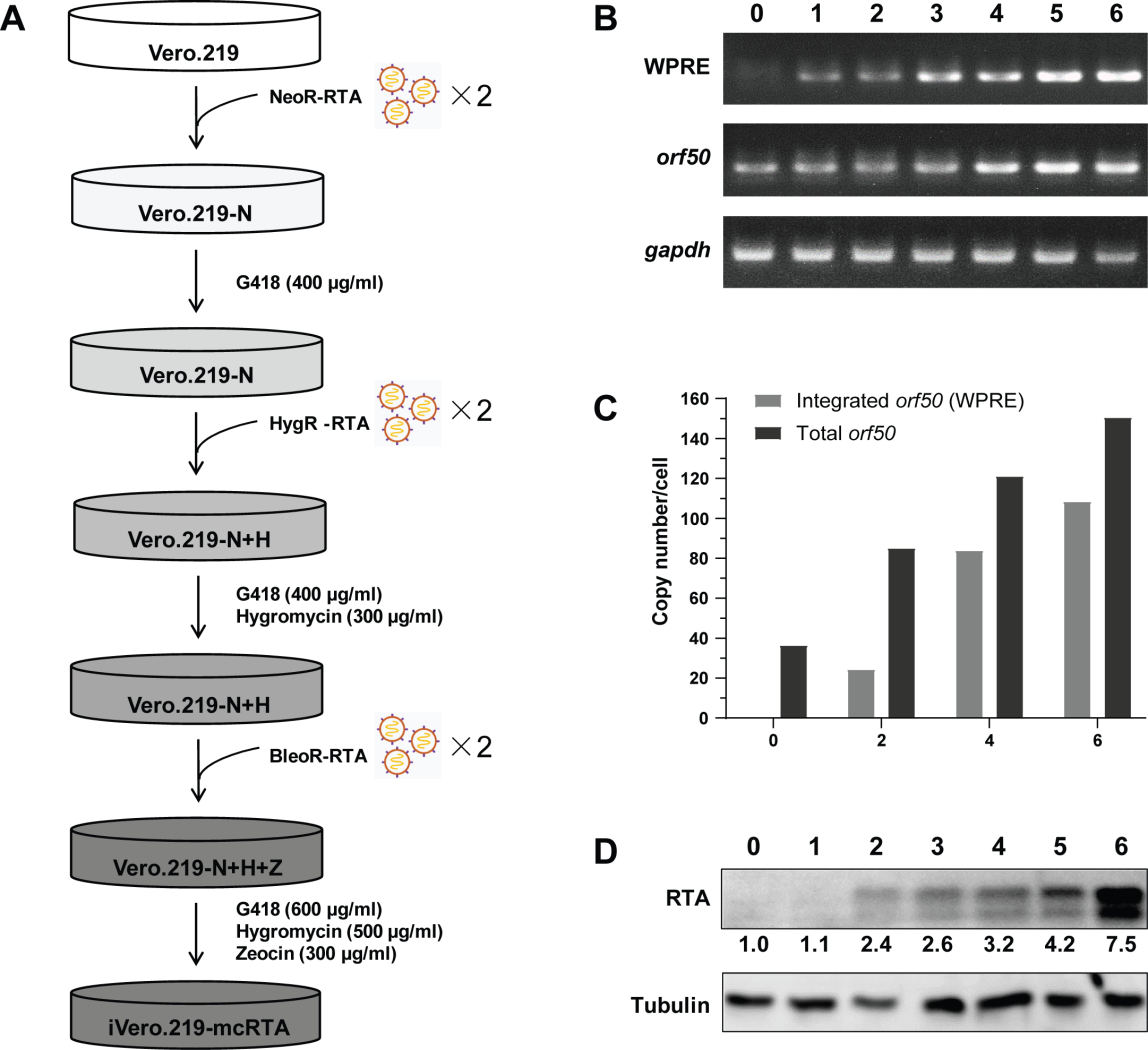
Generation of the iVero.219-mcRTA cell line harboring multicopy DOX-inducible *orf50* genes. **(A)** Schematic overview of the strategy for generating iVero.219-mcRTA. **(B)** PCR detection of lentiviral integration marker WPRE, *orf50*, and internal control *gapdh* using genomic DNA collected before transduction and after each round. **(C)** ddPCR quantification of newly integrated *orf50* copies (*via* WPRE) and total *orf50* copies per cell. **(D)** Western blot analysis of RTA expression with β-tubulin as loading control. Densitometric quantification of RTA bands, normalized to corresponding β-tubulin signals, is presented.

To enable high-titer KSHV production with DOX alone, we engineered Vero.219 cells to harbor multiple copies of the DOX-inducible *orf50* gene. This was achieved through sequential transduction with the recombinant lentiviruses, each carrying the RTA expression cassette and a distinct antibiotic resistance gene, followed by stepwise antibiotic selection with G418 (neomycin), hygromycin, and zeocin, thereby enabling cumulative integration of *orf50* copies. The detailed procedure is outlined in **Figure 2A**, and the resulting polyclonal cell line was designated iVero.219-mcRTA.

To evaluate multicopy lentiviral integration, endpoint PCR was performed to detect WPRE and *orf50* target sequences after each transduction round. A gradual increase in the abundance of both amplicons was observed over successive transductions, with the strongest signal detected after six rounds in the iVero.219-mcRTA cells (**Figure 2B**). To quantitatively assess this integration, qPCR was employed to determine the copy numbers of both newly integrated and total *orf50*. Since the WPRE element is co-integrated with the *orf50* gene from the same transfer vector, its copy number was used as a surrogate for newly integrated *orf50*. The analysis showed a progressive increase in both measures over successive transduction cycles. After the sixth transduction, cells harbored 68 ± 4 newly integrated *orf50* copies per cell, and the total *orf50* copies reached 80 ± 2 (**Supplementary Figure 1**).

To eliminate standard curve dependency and amplification efficiency variability of qPCR, ddPCR was employed for direct absolute quantification of integrated *orf50*. Using *rpp30*, a 2-copy reference gene in Vero cells, as the reference, the results showed that cells harbored approximately 0, 24, 84, and 108 copies of newly integrated *orf50* and 36, 85, 121, and 150 copies of total *orf50* after 0, 2, 4, or 6 rounds of transduction, respectively (**Figure 2C**). While the absolute copy numbers differ between qPCR and ddPCR, both approaches confirm multicopy integration of *orf50*.

To assess RTA protein expression, parental Vero.219 cells and cells subjected to each transduction round were treated with 1.0 µg/mL DOX for 72 h. Western blot analysis showed a transduction-dependent increase in RTA protein levels. Elevated expression was detectable starting from the second transduction and reached a maximum after six rounds, demonstrating that the integrated transgenes were capable of robust, inducible expression (**Figure 2D**).

### 3.3 Efficient lytic reactivation in iVero.219-mcRTA cells upon DOX induction

**Figure 3:**
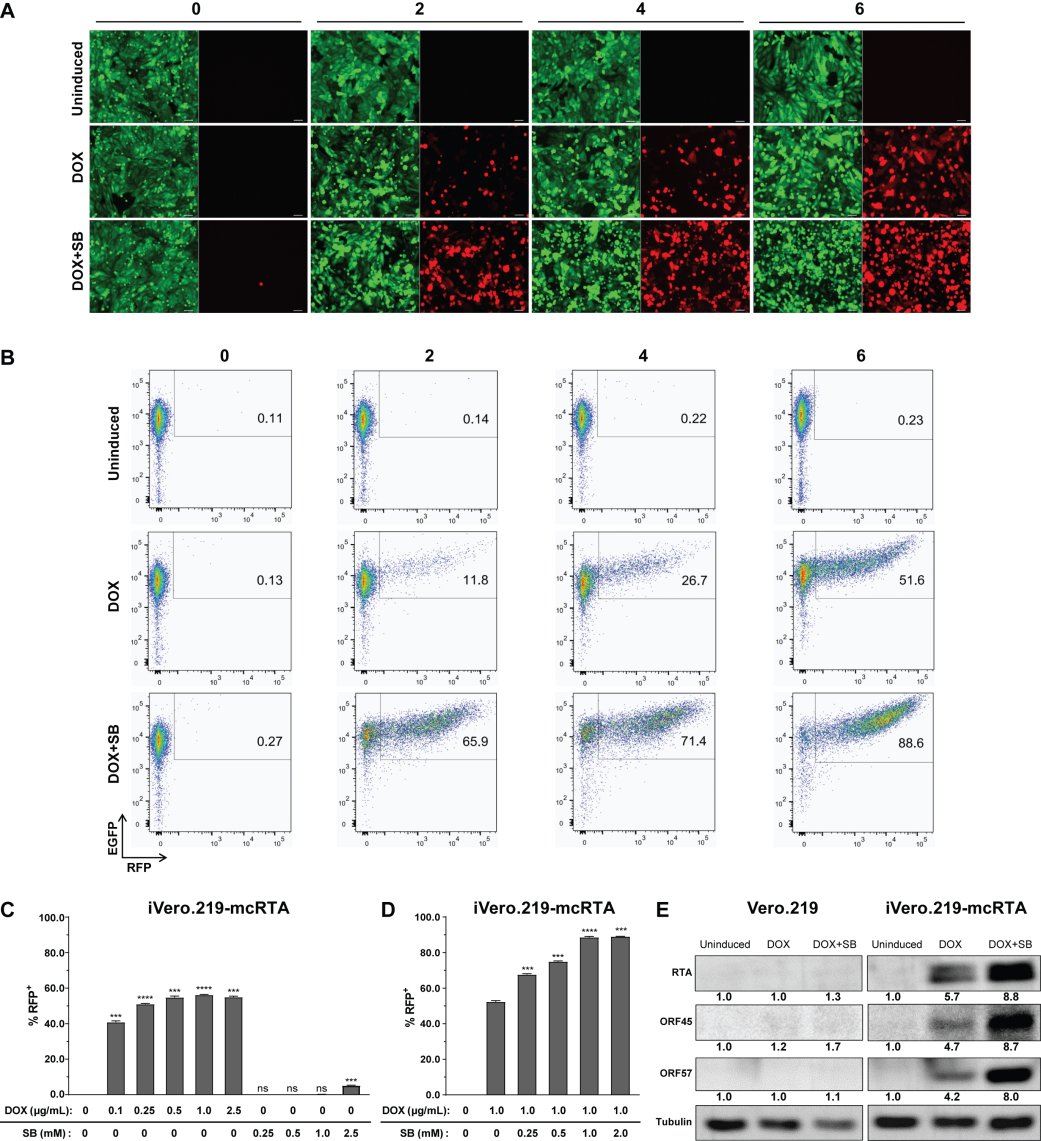
Lytic reactivation of iVero.219-mcRTA cells in response to DOX and SB induction. **(A)** Representative fluorescence microscopy images (20× magnification) of non-transduced Vero.219 cells (0) and cells subjected to 2, 4, or 6 rounds of lentiviral transduction (2, 4, 6). Cells were left uninduced or treated for 72 h with 1.0 µg/mL DOX alone or in combination with 1.0 mM SB. Lytic reactivation was assessed *via* RFP expression. Scale bars: 50 µm. **(B)** Flow cytometry analysis of RFP expression in Vero.219 and iVero.219-mcRTA cells treated as in **(A)**. Values indicate the percentage of RFP-positive cells in the gated population. **(C)** Flow cytometry quantification of RFP-positive iVero.219-mcRTA cells after 6 rounds of transduction. Cells were induced with 0, 0.1, 0.25, 0.5, 1.0, or 2.5 µg/mL DOX, or 0, 0.25, 0.5, 1.0, or 2.5 mM SB. Data represent mean ± SD from three independent experiments. One-way ANOVA with Dunnett’s multiple comparisons test was used to compare each condition against the uninduced control. ***P ≤ 0.001; ****P ≤ 0.0001. **(D)** Flow cytometry analysis of iVero.219-mcRTA cells treated with 1.0 µg/mL DOX plus 0, 0.25, 0.5, 1.0, or 2.0 mM SB. Data are mean ± SD (n=3). Dunnett’s multiple comparisons test was used relative to the DOX-only control. **(E)** Western blot analysis of viral lytic proteins RTA, ORF45, and ORF57 in uninduced, DOX- or DOX plus SB-induced Vero.219 and iVero.219-mcRTA cells.

We next investigated whether latent KSHV in these cells could be reactivated into lytic cycle by DOX-induced RTA expression. Uninduced control cultures were compared to those treated for 72 h with 1.0 µg/mL DOX alone or in combination with 1.0 mM SB. Lytic reactivation was assessed by monitoring RFP reporter expression, a surrogate marker, *via* fluorescence microscopy (**Figure 3A**) and flow cytometry (**Figure 3B**). RFP expression increased with successive transduction rounds, whereas virtually no signal was detectable in uninduced controls. Correspondingly, uninduced cells maintained normal morphology whereas some induced cells exhibited cytopathic effects. With DOX induction alone, the proportion of RFP-positive cells increased progressively from 11.8% after two transductions to 26.7% after four and 51.6% after six. The addition of SB significantly enhanced the efficiency of reactivation mediated by DOX. Treatment with 1.0 µg/mL DOX plus 1.0 mM SB resulted in reactivation rates of 65.9% after two transductions, 71.4% after four, and 88.6% after six. Notably, the relative increase in reactivation efficiency across successive transductions was greater with DOX alone than with the combined treatment.

iVero.219-mcRTA cells that had undergone six rounds of lentiviral transduction were treated with varying concentrations of DOX and SB, and the induction efficiency of KSHV transactivation was quantified using flow cytometry-based detection of RFP (**Figure 3C**). SB alone, at concentrations ranging from 0.25 to 1.0 mM, failed to effectively induce the lytic cycle in these cells. Even at 2.5 mM, induction efficiency remained low, reaching only 5.2%. In contrast, DOX alone robustly induced lytic reactivation across the entire tested concentration range from 0.1 to 2.5 μg/mL. A substantial reactivation efficiency of ~55% was achieved at and above 0.5 μg/mL, with no further significant increase at higher concentrations. To investigate potential synergy, we treated cells with a fixed concentration of 1.0 μg/mL DOX combined with increasing concentrations of SB. The addition of SB further enhanced lytic reactivation efficiency, with a maximum of ~89% achieved at SB concentrations of 1.0 mM and above (**Figure 3D**).

The lytic cycle progression was further validated by Western blot analyses. Consistent with the RFP-based quantification, DOX treatment alone induced the expression of immediate-early proteins RTA and ORF45 and early protein ORF57 in iVero.219-mcRTA cells, an effect further enhanced by DOX and SB co-treatment. In contrast, no significant lytic protein induction was observed in Vero.219 cells under either treatment condition (**Figure 3E**).

### 3.4 High-titer KSHV production from DOX-induced iVero.219-mcRTA cells

**Figure 4:**
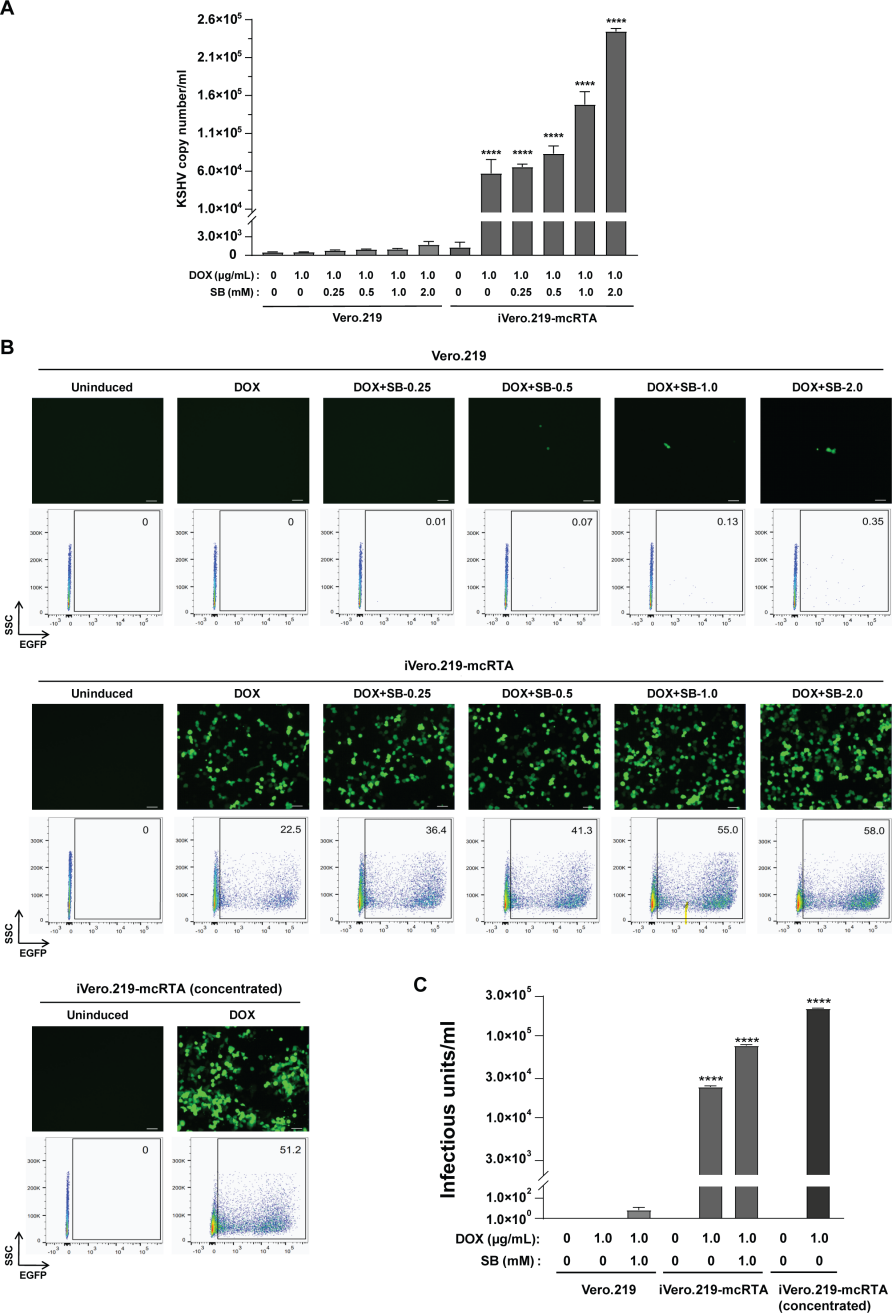
Comparison of infectious virus production from Vero.219 and iVero.219-mcRTA cells following DOX and SB induction. **(A)** Quantification of KSHV genomic DNA in cell-free supernatants by qPCR targeting the *k9* gene. Vero.219 and iVero.219-mcRTA cells were treated as in **Figure 3D**. Data are mean viral copies/mL ± SD (n=3). One-way ANOVA with Tukey’s test was used for comparisons between iVero.219-mcRTA and parental Vero.219 under identical conditions; ****P ≤ 0.0001. **(B)** Infectious virus titration on 293T cells. Supernatants from Vero.219 and iVero.219-mcRTA cells induced with 1.0 µg/mL DOX alone or with varying concentrations of SB were collected and used to infect 293T cells. A 100× concentrated supernatant from iVero.219-mcRTA cells induced with 1.0 µg/mL DOX alone was also tested. Infectivity was assessed by EGFP expression. Top: Representative fluorescence images (20×). Bottom: Corresponding flow cytometry plots; values indicate EGFP-positive cells. **(C)** Infectious titer quantification by Poisson distribution analysis (10–20% EGFP-positive cells). Data are mean ± SD IU/mL (n=3). ****P ≤ 0.0001.

To quantify viral particle production, supernatants were collected from iVero.219-mcRTA cells 72 h post-induction with DOX, either alone or in combination with SB. Released KSHV genomic copies were quantified by qPCR targeting the *k9* gene. For Vero.219 cells, treatment with 1.0 μg/mL DOX plus 2.0 mM SB yielded (1.7 ± 0.4) × 10³ copies/mL in the supernatant. By contrast, iVero.219-mcRTA cells exhibited significantly higher induction efficiency, with DOX alone producing (5.7 ± 1.5) × 10^4^ copies/mL and co-treatment with 2.0 mM SB further elevating yields to (2.4 ± 0.3) × 10^5^ copies/mL, approximately two orders of magnitude higher than those observed in conventional Vero.219 cells (**Figure 4A**).

To evaluate the infectivity of produced virions, supernatants from reactivated Vero.219 and iVero.219-mcRTA cells were used to infect 293T cells. Viral infectivity was assessed 24 h post-infection by measuring EGFP expression *via* fluorescence microscopy and flow cytometry. No infectious KSHV was detected in uninduced control cells of either cell line. Conventional Vero.219 cells treated with 1.0 μg/mL DOX and 2.0 mM SB released progeny virus of low infectivity, resulting in only 0.35% EGFP-positive 293T cells upon infection. In contrast, iVero.219-mcRTA cells treated with DOX alone produced substantial infectious virions, yielding 22.5% EGFP-positive cells. SB supplementation further enhanced production, resulting in 55.0% and 58.0% EGFP-positive cells with 1.0 mM and 2.0 mM SB, respectively (**Figure 4B**). For quantitative comparison, these values were converted to infectious units (IU) based on cell density. Conventional Vero.219 cells induced with 1.0 μg/mL DOX plus 1.0 mM SB yielded only 6.7 ± 4.7 IU/mL. In contrast, iVero.219-mcRTA cells produced titers of (2.4 ± 0.1) × 10^4^ IU/mL with DOX alone, which increased to (7.5 ± 0.2) × 10^4^ IU/mL when 1.0 mM SB was added (**Figure 4C**). These data demonstrate that iVero.219-mcRTA yields infectious titers more than 4 orders of magnitude above those of conventional Vero.219 cells under equivalent induction conditions.

To achieve even higher KSHV titers from DOX induction alone, supernatant from induced iVero.219-mcRTA cells was concentrated 100×. Infection of 293T cells with the concentrated supernatant achieved 51.2% EGFP-positive cells, corresponding to a titer of (2.1 ± 0.1) × 10^5^ IU/mL (**Figures 4B, C**). Thus, high-titer KSHV can be robustly produced by DOX induction alone, eliminating the necessity for supplementary HDACi such as SB.

## 4 Discussion

In this study, we generated the novel iVero.219-mcRTA cell line as an inducible system that supports robust KSHV lytic replication and high-titer virion production. A key feature of this model is the genomic integration of multiple copies of the KSHV *orf50* gene, which encodes the master lytic switch protein RTA. This was achieved through optimized sequential lentiviral transduction, in which each transfer vector contained a DOX-inducible *orf50* expression cassette together with a distinct antibiotic-resistance marker, enabling progressive enrichment of transduced cells. As a result, multicopy integration of the *orf50* transgene was achieved, with approximately 150 total copies per cell, as determined by ddPCR. This strategy directly addresses a recognized limitation in KSHV reactivation models: the frequent inadequacy of RTA expression to overcome host epigenetic silencing, which often restricts efficient lytic cycle initiation (Purushothaman et al., 2015). The iVero.219-mcRTA system thus offers an advantage over the parental Vero.219 cell line, which depends on exogenous RTA expression or chemical induction, and over other adherent models such as iSLK.219 and iTIME.219, which typically rely on random integration of a single *orf50* transgene, an approach that often results in constrained RTA expression and incomplete reactivation from epigenetic silencing (Myoung and Ganem, 2011; DeCotiis et al., 2017; Dollery et al., 2021). The discrepancy in *orf50* copy number between qPCR and ddPCR may have resulted from the inherent methodological differences: qPCR-based relative quantification employed *gapdh* assuming 2 copies per cell, an assumption potentially compromised in aneuploid Vero cells, whereas ddPCR enables direct absolute quantification that eliminates standard curve dependency and amplification efficiency variability, using *rpp30* as a validated 2-copy reference (Zhang et al., 2025).

The iVero.219-mcRTA system is based on the Lenti-X Tet-One platform, in which *orf50* expression is controlled by the highly sensitive yet tightly regulated *P*_TRE3G_ promoter, a system also employed in iSLK and iTIME models (Loew et al., 2010). This design enables exceptionally stringent DOX-dependent control of RTA expression, a crucial attribute for studies of lytic reactivation. The iVero.219-mcRTA model exhibits minimal spontaneous reactivation (0.23%) in the absence of DOX (**Figure 3B**), in stark contrast to the characteristically high spontaneous reactivation rates observed in PEL-derived B-cell lines such as BCBL-1 and BC-3, where high background complicates experimental interpretation (Renne et al., 1996). The efficiency of the system is evident from DOX induction alone triggering lytic cycle entry in 51.6% of iVero.219-mcRTA cells, a rate that could be synergistically enhanced to 88.6% with SB co-treatment. The reactivation mechanism in iVero.219-mcRTA differs fundamentally from that in PEL cells. In iVero.219-mcRTA, reactivation is initiated by direct, controlled expression of RTA from integrated transgenes, whereas in PEL cells, chemical inducers such as TPA or HDACi act through pleiotropic pathways that can activate endogenous viral promoters or cellular signaling cascades less specifically (Sun et al., 1999; Dollery et al., 2014).

With respect to viral production, the iVero.219-mcRTA cell line represents a substantial improvement over its parental counterpart. Conventional Vero.219 cells typically generate low viral titers, often below 1 × 10^4^ IU/mL, even with potent chemical induction using TPA/SB combinations. In contrast, iVero.219-mcRTA cells produced substantial infectious virus with DOX alone, reaching 2.4 × 10^4^ IU/mL, and the titer increased to 7.5 × 10^4^ IU/mL upon SB addition. The 100× concentrated supernatant from DOX-induced cultures reached a titer of 2.1 × 10^5^ IU/mL, meeting the requirements of standard functional studies. The less-than-proportional increase in titer following concentration, corresponding to a recovery efficiency of approximately 9%, may reflect a combination of expected technical losses during processing, virus aggregation induced by high-speed centrifugation, and potential damage to viral integrity, such as envelope disruption caused by the shear forces.

The capacity to generate high-titer viral preparations in the absence of HDACi like SB represents a particular advantage. SB and related short-chain fatty acid HDAC inhibitors exert pleiotropic effects, including widespread histone hyperacetylation, altered chromatin architecture, and modulation of immune signaling and non-histone targets, that can introduce significant confounding effects in subsequent infection experiments (Purushothaman et al., 2015; Silberman et al., 2016; Hopcraft et al., 2018). The DOX-only system reduces these potential confounding variables associated with chemical inducers. Our preliminary data indicate that iVero.219-mcRTA cells maintain stable production capacity through routine subculture and freeze-thaw cycles under continuous antibiotic selection. Future work will include long-term stability testing over extended passages and batch consistency evaluation.

We acknowledge several limitations of this model. Vero cells are characterized by aneuploid karyotype and genetic heterogeneity (Osada et al., 2014; Andreani et al., 2017). Six rounds of transduction may increase the risk of insertional mutagenesis and clonal variation, and polyclonal populations can undergo phenotypic drift during long-term culture. Consequently, strict quality control and precautions are essential, such as using low-passage cell stocks (P5–P15), regularly validating *orf50* copy number and lytic inducibility, and performing single-cell cloning for highly reproducible experiments.

In addition, the epigenetic landscape of Vero cells differs from that of primary human targets of KSHV, such as B cells and endothelial cells (Ganem, 2010). A key limitation is that Vero cells are deficient in type I interferon production (Desmyter et al., 1968; Emeny and Morgan, 1979; Chew et al., 2009), a feature not seen in either iSLK or iTIME cells, which retain intact innate immune pathways. This constraint limits the applicability of our system, as iVero.219-mcRTA is unsuitable for studies requiring intact interferon or innate immune responses. Nonetheless, the iVero.219-mcRTA represents a novel Vero-based cell line with multicopy RTA integration enabling reliable, DOX-only high-titer production of rKSHV.219, serving as a unique complement to endothelial-based lines such as iSLK.219 and a robust, valuable addition to the KSHV research toolkit.

## Data Availability

The original contributions presented in the study are included in the article/**Supplementary Material**. Further inquiries can be directed to the corresponding authors.
