# Supplementary material for: Generation of iVero.219-mcRTA: a doxycycline-inducible high-titer KSHV producer cell line with multicopy orf50 integration
Source: Front Cell Infect Microbiol. 2026 Apr 13;16:1760149. doi: 10.3389/fcimb.2026.1760149 (PMC13111134; doi:10.3389/fcimb.2026.1760149)
Supplement: Supplementary file 1 [file SupplementaryFile1.doc]

Supplementary Table 1: Oligonucleotides used in this study

| Oligonucleotide | Sequence (5’ to 3’) |
| --- | --- |
| BleoR-F | ATATTAGTTAACGCCACCATGGCCAAGTTGACC |
| BleoR-R | AATCACACGCGTTCAGTCCTGCTCCTC |
| MCS-F | GATCCGGAGCGGCCGCTTTCGAAATCGATTGGG |
| MCS-R | AATTCCCAATCGATTTCGAAAGCGGCCGCTCCG |
| ORF50-F | GCGTGGATCCTCACGGAGAGGATCTTAT |
| ORF50-R | TATAGCGGCCGCTCAGTCTCGGAAGTAATT |
| qPCR-WPRE-F | GGCACTGACAATTCCGTGGT |
| qPCR-WPRE-R | AGGGACGTAGCAGAAGGACG |
| qPCR-RTA-F | GCCGGTGTCTCAAGAGCTG |
| qPCR-RTA-R | CGCAATGCGTTACGTTGTTG |
| qPCR-GAPDH-F | TCACTGCCACCCAGAAGACT |
| qPCR-GAPDH-R | ATGACCTTGCCCACAGCCTT |
| qPCR-K9-F | GTCTCTGCGCCATTCAAAAC |
| qPCR-K9-R | CCGGACACGACAACTAAGAA |
| ddPCR-RTA-F | CGCAATGCGTTACGTTGTTG |
| ddPCR-RTA-R | GCCCGGACTGTTGAATCG |
| ddPCR-RTA-probe | 6FAM-ACCTGTGCCCCCTCTTCGACACC-BHQ1 |
| ddPCR-WPRE-F | CATTGCCACCACCTGTCA |
| ddPCR-WPRE-R | CGACAACACCACGGAATT |
| ddPCR-WPRE-Probe | 6FAM-AGGCAGGCGGCGATGAGT-BHQ1 |
| ddPCR-RPP30-F | CAGACTTGGACGTGCGAG |
| ddPCR-RPP30-R | CCGCGGTCTCCACAAGTC |
| ddPCR-RPP30-Probe | HEX-CTGACCTGAAGGCTCTGCGCG-BHQ1 |


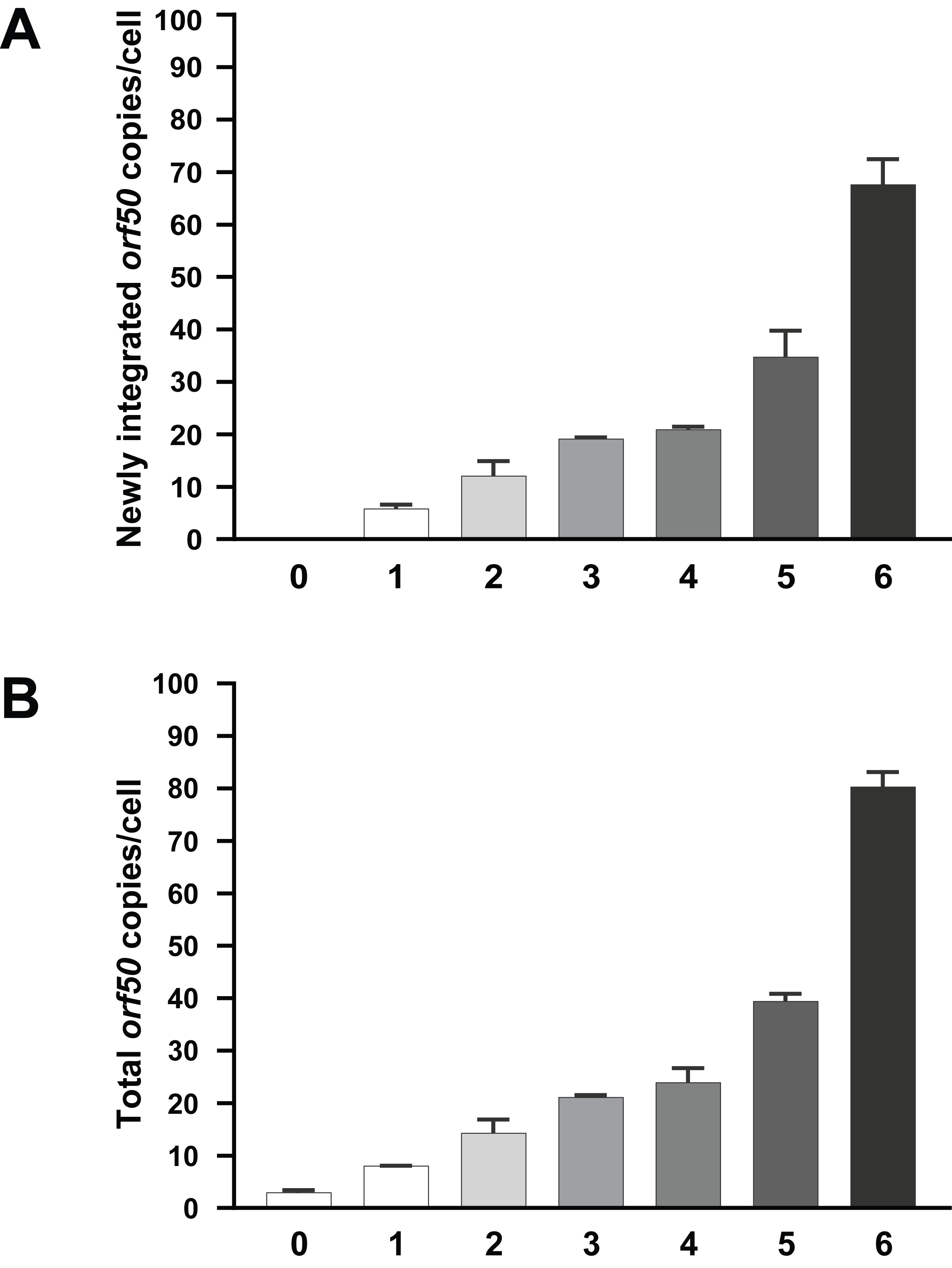


Supplementary Figure 1. qPCR quantification of (A) newly integrated *orf50* copies (*via* WPRE) and (B) total *orf50* copies per cell in cells collected before transduction (0) or after each round of transduction (1–6). Data are presented as mean ± SD (n=3).
